# Non-Specific Immunity Associated Gut Microbiome in *Aristichthys nobilis* under Different Rearing Strategies

**DOI:** 10.3390/genes12060916

**Published:** 2021-06-14

**Authors:** Jianming Yuan, Zhijian Wang, Bo Wang, Huiqing Mei, Xuliang Zhai, Zhenhua Zhuang, Maoshan Chen, Yaoguang Zhang

**Affiliations:** 1 Key Laboratory of Freshwater Fish Resources and Reproductive Development, Ministry of Education, Southwest University, Chongqing 400715, China; yuanjianming84@163.com; 2 Chongqing Fisheries Technology Promotion Station, Chongqing 400020, China; wangzj@swu.edu.cn (Z.W.); wangbo@cqagri.gov.cn (B.W.); meihuiqing6469@163.com (H.M.); zhaizige@sohu.com (X.Z.); 3 Chengdu Life Baseline Technology, Chengdu 610041, China; zhuangzhenhua@genebang.com; 4 Australian Centre for Blood Diseases, Central Clinical School, Monash University, Melbourne, VIC 3004, Australia; maoshanchen@gmail.com

**Keywords:** *Aristichthys nobilis*, 16S rRNA gene, *Lactic acid bacteria*, non-specific immunity

## Abstract

To understand the intestinal microbial diversity and community structure of bighead carp (*Aristichthys nobilis*) under different feeding strategies, 39 fish from three groups (A: 9 fish, natural live food only; B: 15 fish, natural live food + fish formulated feeds; C: 15 fish, natural live food + fish formulated feed + *lactic acid bacteria*) were obtained for the high throughput 16S rRNA gene sequencing. We first examined five non-specific immunity indications of the carp—lysozyme (LZM), catalase (CAT), glutathione reductase (GR), glutathione peroxidase (GSH-PX), and superoxide dismutase (SOD). Interestingly, the composition of gut microbiota and related non-specific immune indices were affected by the feeding treatment of the bighead carp. Notably, all enzyme activity indexes were significantly different (*p* < 0.01) in the spleen and three enzyme activity indexes (LZM, GSH-PX, and SOD) had significant differences in the hepatopancreas (*p* < 0.001) of the carp from the three groups. The 16S rRNA gene sequencing showed higher diversity in groups B and C. Compared to group A, the relative abundance of *Actinobacteria* increased significantly and the relative abundance of *Proteobacteria* and *Firmicutes* decreased significantly in groups B and C at the phylum level. Functional analysis revealed the association between non-specific immune indicators and import genera in the hepatopancreas and spleen of bighead carp. This study provides new insights into the gut microbiomes and non-specific immune of bighead carp.

## 1. Introduction

The bighead carp (*Aristichthys nobilis*) is a cyprinid fish that is native to South China and mainly feeds on zooplankton and phytoplankton in natural waters [1,2]. As a well-known economic fish, the global annual production of bighead carp is about ~10 million tons per year. In recent years, with increasing demand for bighead carp consumption, there has been wide concern about high-density pond farming of bighead carp [3]. In addition, feeding bighead carp with various formulation feeds to increase yield has become popular and acceptable [4]. However, due to the large-scale use of fertilizers and various fish feeds, the fish breeding environment has gradually deteriorated, resulting in increased stress factors and decreased autoimmune capacity, which can cause chronic diseases of the fish, and is one of the most pressing concerns in modern fish farming [5].

Lactic acid bacteria are natural active microorganisms that have regulatory effects on the internal environment of the gastrointestinal tract of animals [6]. They were reported to have the ability to improve the ecological environment of intestinal microbes through biological antagonisms, strengthen the barrier layer of intestinal epithelial cells, and improve the immune function of animals [7]. Previous studies on lactic acid bacteria in feed research showed that lactic acid bacteria can improve the intestinal environment of livestock and poultry, inhibit the growth and reproduction of harmful bacteria, and enhance the disease resistance in pigs and chickens [8]. Some studies were conducted to investigate the association between lactic acid bacteria and fish farming. For example, Son et al. showed improved weight gain rate and feed efficiency of groupers after adding lactobacillus into their feeds for 4 weeks [9]. Aly et al. found that the weight and survival rate of tilapia greatly improved after lactic acid bacteria were applied to their feeds [10]. Suzer et al. applied lactobacillus to black snapper at the development stage of larva and found that the growth performance and digestive enzyme activity of larva improved, especially when lactobacillus was added to live feeds or water [11]. However, little is known about the effects of lactic acid bacteria in bighead carp.

Innate immunity usually plays a very important role in combating microbial infection for all animals [12], and in general, the lysozyme (LZM), catalase (CAT), glutathione reductase (GR), glutathione peroxidase (GSH-PX), and superoxide dismutase (SOD) are key indices representing the non-specific immunity of animals in vivo [13]. LZM is a widely distributed cationic enzyme, which can destroy and eliminate foreign matter invading the body [14]. CAT and SOD are the main antioxidant enzymes of the oxidation resistance defense system in fish [14]. GSH-PX is an enzyme that prevents damage to cellular membranes and participates in the immune response of animals, which can directly reflect the body’s antioxidant capacity and immune level [15]. Thus, these indicators can be used to show the immune ability of fish and increase of the immune enzyme activity can enhance the immune system of fish.

Intestinal microbiota, as an important part of the host body, also plays an important role in fish health [16]. The dominant microorganisms in the fish gut are usually bacteria [17], which are mainly composed of aerobic bacteria and facultative anaerobic bacteria, among them, three core microorganisms were dominant: *Firmicutes*, *Proteobacteria*, and *Bacteroidetes* [18,19]. However, our knowledge about the intestinal flora of filter-feeding fish fed with formulated feeds is still poor. Traditional microbial research methods mainly rely on microbial culture technology, which can only detect less than 1% of microorganisms in nature [20]. Compared to traditional microbial identification and analysis methods, in the last ten years, the 16S rRNA high-throughput sequencing technology, a new generation sequencing method that possesses high accuracy and sensitivity [21,22], was widely used in the intestinal microbial studies, can greatly improve the depth and breadth of the analysis of microbial diversity in samples, and can improve our understanding of the structure and function of intestinal microflora. Considering the influence of *lactobacillus* on the fish gut bacterial community [8,9,10,11], using 16S rRNA sequencing technology to explore how formulated feeds change the intestinal microbial composition of bighead carp would be of certain interest. The aims of the present study include the investigation of the intestinal microbiome differences under natural feeding and fodder feeding, specially focusing on the effect of lactic acid bacteria on the intestinal flora and non-specific immune enzyme activity of bighead fish. The results will improve our understanding of the gut microbiome of bighead carp and will provide new ideas to study the fish intestinal flora.

## 2. Materials and Methods

### 2.1. Experimental Designs and Fish Management

All experimental bighead carp species were cultivated from the same batch of larvae from the Aquatic Products Research and Development Center of Jingzhi (Chongqing) Smart Agriculture Company. The experiment was carried out in eight outer pond cages (2 m × 2 m × 1.5 m) with 2.5 m of average water depth. Three groups of bighead carp were fed by different materials, including group A (2 parallel)—the control group fed with the ingested plankton in the water; group B (3 parallel) mainly fed with the artificial diets; and group C (3 parallel) fed with artificial feed plus lactic acid bacteria. Thus, a total of eight cages were used for these three groups and they were transferred from Jingzhi Smart Agriculture Company to the Beibei District Fish Base of the Southwest University (Chongqing). The artificial feed for this research uses the special feed for bighead carp from Aolong Kexiong Feed Co., Ltd. Chongqing, China. The biochemical composition of the artificial feed consisted of moisture (8.96%), crude protein (31.82%), crude fat (6.86%), crude ash (9.30%), crude fiber (4.91%), calcium (0.86%), and total phosphorus (1.39%). The culture experiment lasted for 8 weeks (56 days). Fish from groups B and C were fed twice a day at 8:30 a.m. and 5:30 p.m. The pond was regularly disinfected, bottomed, and oxygenated, according to normal aquaculture production management, to ensure water quality was under optimal conditions.

### 2.2. Sample Collection and Pre-Processing

At the end of the trial, fish were carefully captured by the nets in order to avoid external physiological stimulation. Five bighead carp with similar weight were randomly selected from each cage and anesthetized with MS-222 (Syndel, Ferndale, WA, USA) on ice. A total of 39 fish (group A: 9; group B: 15; group C: 15) were dissected. After the intestines were cut and taken out, the outer wall of the intestinal tube was wiped with 75% ethanol and then rinsed with sterile saline. The intestinal contents were evenly collected by 5 mL EP tubes and immediately placed in liquid nitrogen for 6 h. All samples were transported to the laboratory and stored at −80 °C refrigerator in time until DNA extraction.

Fish hepatopancreas and spleen were separated in order to determine related enzyme activity. The hepatopancreas and spleen were blotted with a filter paper to absorb the surface water and placed in sterile 1.5 mL centrifuge tubes. After the samples were centrifuged at 10,000 rpm for 15 min, the supernatant was collected and then stored at −20 °C to determine the enzyme activity. The experiments were performed in accordance with the Regulations for the Administration of Affairs Concerning Experimental Animals of China and the protocols applied in the present study were approved by the Ethics Committee of Southwest University (approved IACUC NO: 20200720-1).

### 2.3. Non-Specific Immunity Related Enzyme Index Determination

Lysozyme (LZM), catalase (CAT), glutathione reductase (GR), glutathione peroxidase (GSH-PX), superoxide dismutase (SOD) activities were measured by using commercial kits according to the manufacturers’ instructions (Nanjing JianCheng Bioengineering Institute, Nanjing, China).

### 2.4. DNA Extraction, PCR, and Sequencing

For bacterial 16S ribosomal RNA gene sequencing, microbial DNA was extracted from all 39 fish gut contents using the Qiagen Stool DNA Kit (Norcross, GA, USA) following the manufacturer’s protocol. The V4–V5 region of the bacterial 16S ribosomal RNA gene was amplified using the primers 515F (5′-GTGCCAGCMGCCGCGG-3′) and 907R (5′-CCGTCAATTCMTTTRAGTTT-3′), where the barcode was an eight-base sequence unique to each sample. The PCR experiment was carried out as described [23]. All samples were carried out in accordance with standard experimental condition in triplicates. The PCR products were then mixed and run on a 2% agarose gel. After the targeting regions were cut and recovered using the AxyPrepDNA Gel Recovery Kit (AXYGEN, Union City, CA, USA), they were quantified by Qubit^®^ 3.0 (Invitrogen, Waltham, MA, USA) and mixed equally with barcodes. Then, the mixture was used to prepare the DNA pair-end library following the Illumina library preparation protocol and sequenced on the Illumina MiSeq platform in Shanghai BIOZERON (Shanghai, China) with paired-end 250 strategy. All raw sequencing data generated by this study can be accessed from the NCBI short read archive (SRA) platform under the accession number PRJNA695396.

### 2.5. Statistical and Bioinformatics Analysis

Raw FASTQ files were demultiplexed using in-house Perl scripts, according to the barcode sequences of all samples, and cleaned with the following criteria: (i) the 250 bp reads were truncated at any site receiving an average quality score <20 over a 10 bp sliding window and truncated reads shorter than 50 bp were discarded; (ii) exact barcode match, reads containing ambiguous characters were removed. We removed the reads that were not assembled [24]. After data cleaning, we identified operation taxonomic units (OTUs) for each sample using UPARSE (V7.1) with 97% similarity [25]. UCHIME was applied to identify and remove chimeric sequences based on both reference database and de novo assembly analysis [26]. The phylogenetic affiliation analysis of each 16S rRNA gene sequences was introduced by RDP Classifier against the SILVA (SSU115) 16S rRNA database with a confidence threshold of 70% [27,28].

The rarefaction analysis based on mothur (v.1.21.1) was conducted to identify the diversity indices [29], including the Chao, Shannon, and Simpson diversity indices. The unweighted pair-group method based on Bray–Curtis dissimilarity was used to calculate the distance between two samples, subsequent β-diversity analysis, and visual statistical analysis were performed with the data. Principal co-ordinate analysis (PCoA) was performed using the community ecology package and R-forge, and the results were visualized using the Vegan 2.0 package. Linear discriminant analysis effect size (LEfSe) analysis used to identify differentially abundant bacteria taxa between groups (A vs. B and A vs. C) according to the relative abundance and the *p*-value less than 0.05 was considered as significant difference [30]. Kruskal–Wallis sum-rank test was applied to examine the changes and differences between groups, followed by the LDA analysis to determine the size effect of each distinctively abundant taxa [31]. The enzyme activity data were presented in mean ± SD. We used the ‘boxplert’ function in the R for data statistics. One-way ANOVA analysis was used to assess the differences in bighead carp enzyme activity among the different groups. Once the significant difference was found, Dunn’s Multiple Test was used to perform the multiple comparisons. For correlation analysis, the relationships between genera OUT abundance and enzymatic activity were investigated separately through Pearson correlation calculation to explore the potential metabolic functions; the six genus abundance data were log2 transformed before Pearson analysis to reduce the effect of extremely high values, and the *p*-value less than 0.05 was considered a significant pairwise relationship.

## 3. Result

### 3.1. Biochemical Parameters Related to Enzyme Activity

To understand the effect of lactic acid bacteria on the intestinal flora and non-specific immune enzyme activity of bighead carp, we bred a total of 400 fish with similar weight in eight cages for three groups—group A (two parallel, fed with the ingested plankton in the water); group B (three parallel, fed with the artificial diets); and group C (three parallel, fed with the artificial diet plus lactic acid bacteria). The fish were maintained in the pond for two months and the characteristics can be found in Table 1. It is notable that compared to group A, bighead carp from groups B and C had significant increase of weight and visceral body ratio after two-month breeding. Then, the intestines were cut and taken out for subsequence analyses.

We first examined the enzyme activities of bighead carp form the eight cages (Table S1) and statistical analysis showed that feeding regime can significantly affect the enzymatic activities of the fish visceral tissue. Three indicators of enzymatic activity (LZM, GSH-PX, and SOD) showed significant differences (*p* < 0.001) in hepatopancreas (Table 1). The LZM activity of bighead carp was significantly lower (*p* < 0.001) in groups B and C, compared to group A. The GSH-PX activity was significantly higher in groups A and B, compared to group C, indicating that lactic acid bacteria may affect the level of enzyme activity in hepatopancreas. In addition, we found that bighead carp of group B had the highest SOD activity, and that group A had the lowest SOD activity (*p* < 0.001). Overall, artificial aquaculture practices can significantly increase the SOD activity. No significant (*p* > 0.05) differences in CAT and GR activities were observed in these groups. Interestingly, we found these five indicators with significant difference in the spleen (Table 1). Contrary to the results of hepatopancreas, the activity of LZM was significantly higher (*p* < 0.01) in groups B and C, compared to group A in the spleen. Higher CAT activity was found in group B and lower GR activity was found in group A. In addition, the GSH-PX activity was significant higher (*p* < 0.001) in group A compared to B and C.

### 3.2. Gut Bacterial Diversity

**Figure 1:**
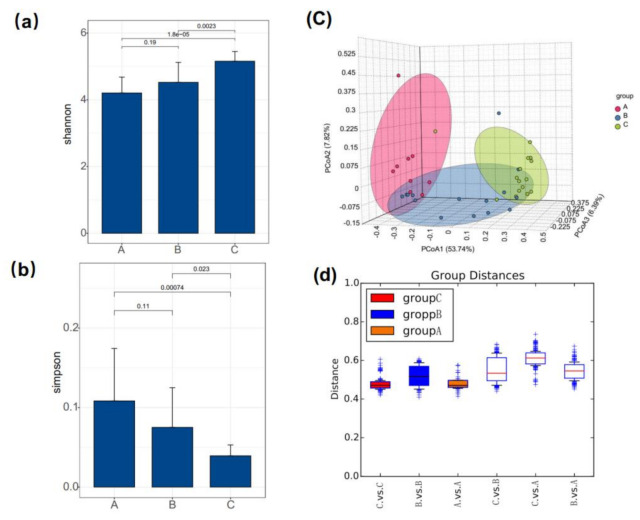
α-diversity Shannon (**a**) and Simpson (**b**) indexes based on average OTUs of bighead carp from the three groups. The values on the horizontal line represent the *p* value of the Wilcoxon signed-rank test. Error bars indicate the SD values. (**c**) Principal co-ordinate analysis (Bray–Curtis distance) of β-diversity. PCoA1, PCoA2, and PcoA3 represent the top three principal coordinates that, together, captured most of the diversity. (**d**) Boxplot for comparisons of intra-group and inter-group based on group distances. A, nature live food only; B, nature live food + fish feed; C, nature live food + fish feed + lactic acid bacteria.

After data cleaning of the sequencing data, a total of 2,360,939 high-quality bacterial reads were obtained for the 39 samples, with an average of 60,654 (min: 32,680, max: 74,751) reads per sample (Table S2). Then, the OTUs were taxonomically classified into different levels using the RDP classifier at a 97% confidence. Based on four different indicators (the numbers of OTU observed, Chao, Shannon, and Simpson indexes) we observed that bighead carp had the highest biodiversity (the highest OUTS, Chao, and Shannon, the lowest Simpson indexes) in group C among the three groups (Table 2). Notably, compared with group B all the four indicators changed in group C, indicating that lactic acid bacteria may affect the diversity of intestinal microbiome in bighead carp. Further, the Shannon and Simpson indices showed significant difference in group B vs. C and group A vs. C (Figure 1a,b). Interestingly, no significant difference of Shannon and Simpson indices was observed between groups A and B (*p* > 0.05). We next performed the principal co-ordinate analysis (PCoA) based on Bray–Curtis distance and clearly separated the bighead carp of the three groups (Figure 1c). These results suggest that the microbiota profiles of carp might be modified by the artificial diet on the OUT level, but not significant (Table 2). In addition, by analyzing the intra-group and inter-group distances of all samples, we found that group B had a larger intra-group distance compare to groups A and C, at the same time, there was a larger intergroup distance between group B and group C (Figure 1d).

### 3.3. Gut Bacterial Composition

**Figure 2:**
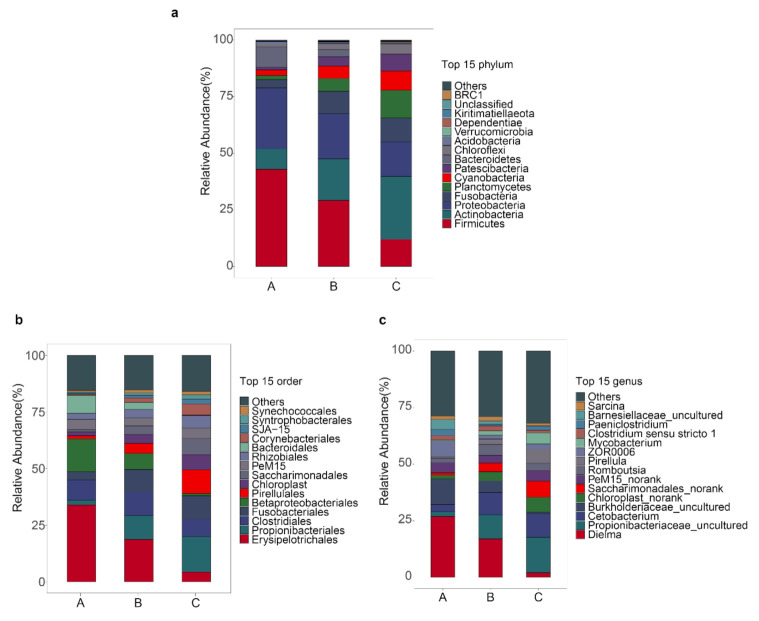
Global composition of bacteria at different taxonomic levels. (**a**) Phyla level (**b**) order level (**c**) genus level. A, nature live food only; B, nature live food + fish feed; C, nature live food + fish feed + lactic acid bacteria. Only the top 15 organisms are presented.

We analyzed the bacteria community composition at three different taxonomic levels. First, we found that at phylum level phyla *Firmicutes*, *Actinobacteria*, *Proteobacteria,* and *Fusobacteria* were the dominant bacteria in all three groups (Table S3). Compared to group A, *Firmicutes* and *Proteobacteria* were significantly decreased but *Actinobacteria* and *Fusobacteria* were significantly increased in both groups B and C (Table S4). Notably, the minor bacteria phylum *Planctomycetes* showed higher expression (Table S4, *p* < 0.05) in group C. At the order level, we found the predominant orders of microorganism species of the bighead carp were *Erysipelotrichales*, *Propionibacteriales*, *Clostridiales*, *Fusobacteriales,* and *Betaproteobacteriales* (Figure 2b). Interestingly, the relative abundance of order *Erysipelotrichales* significantly decreased after the bighead carp were fed by the artificial diet while the *Propionibacteriales* and *Pirellulales* increased (Table S4). At the genus level (Figure 2c), we found that genus *Dielma* and *Burkholderiaceae*_uncultured dominated the intestinal microbiome in both groups A and B. Compared with group A, group B had one more dominant bacteria species *Cetobacterium*. However, the genus *Dielma* OUT was significant lower in group C, compared to groups A and B (Table S4). The dominant bacteria species in group C included Propionibacteriaceae_uncultured (phylum *Bacteroidetes*, order *Propionibacteriales*), Chloroplast norank (phylum *Cyanobacteria*, order *Chloroplast*) and Saccharimonadales_norank (phylum *Bacteroidetes*, order *Saccharimonadales*). In all three groups, we found that some sequences (approximately 30%) were assigned to unknown bacteria.

### 3.4. Differential Bacterial Composition

**Figure 3:**
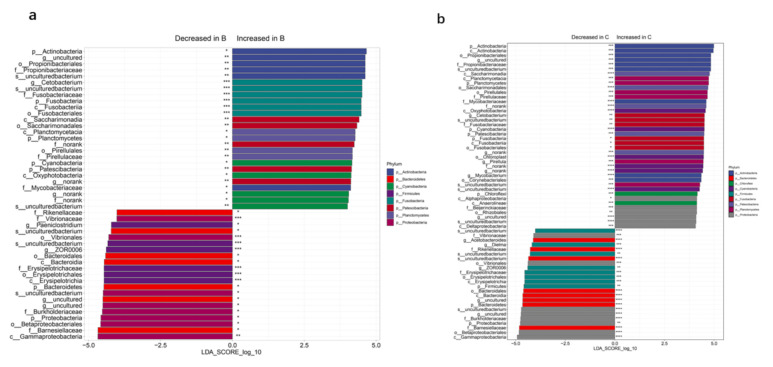
Bacteria taxa differentially expressed in fish intestinal microbiota of the three groups of bighead carp. (**a**) Differentially expressed bacteria identified in group B (nature live food + fish feed, *n* = 15) compared to group A (nature live food only, n = 9). (**b**) Differentially expressed bacteria identified in group C (nature live food + fish feed + lactic acid bacteria, *n* = 15). The histograms represent the linear discriminant analysis (LDA) score computed for taxa differentially abundant between the different groups. *** *p* < 0.001; ** *p* < 0.01; * *p* < 0.05.

We next identified the significant differences in bacteria communities between the three different groups using LEfSe. Compared to group A, we identified 47 bacteria species differentially expressed (*p* < 0.05) in group B, including 21 downregulated and 26 upregulated (Table S4). The overexpressed bacteria in group B included *Fusobacteria*, *Actinobacteria*, *Planctomycetes*, *Patescibacteria*, and *Cyanobacteria* while the downregulated bacteria included *Bacteroidetes* and *Proteobacteria* at phylum level (Figure 3a). After the lactic acid bacteria was added to the feeds for bighead carp, we identified 64 bacteria species differentially expressed (*p* < 0.05) in group C (Figure 3b), including 24 downregulated (e.g., *Proteobacteria*, *Firmicutes*, *Bacteroidetes*) and 40 upregulated (e.g., *Chloroflexi*, *Cyanobacteria*, *Actinobacteria*, *Planctomycetes*, *Patescibacteria*, and *Fusobacteria*). Notably, the phylum *Chloroflexi* was the only bacteria significantly increased in group C compared to group B, which indicated that lactic acid bacteria may have increase the abundance of *Chloroflexi* in bighead carp gut flora. Compared with group B, *Firmicutes* was found to be decreased in group C.

### 3.5. Functional Analysis

**Figure 4:**
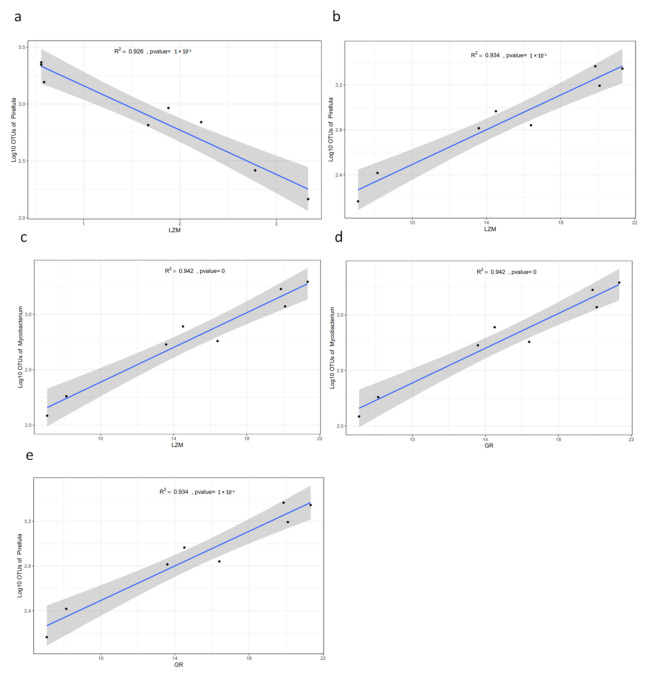
Scatter plot (**a**–**e**) showing the top 5 Pearson correlation coefficient between genus taxonomy and enzyme activities. a, in hepatopancreas; b–e, in the spleen. *X*-axis, enzyme activity indicator; *Y*-axis, log10 value of OUT abundance. LZM, lysozyme; GR, glutathione reductase.

In order to explore the potential non-specific immune function related gut microbes in bighead carp, based on the LEfSe results we manually screened six significant differential expressed genera in group C or group B compared to group A, including *Cetobacterium*, ZOR0006, *Dielma*, *Acetobacteroides*, *Mycobacterium*, and *Pirellula*. Pearson correlation analysis revealed that the genera *Acetobacteroides*, *Cetobacterium*, *Dielma*, *Mycobacterium*, and *Pirellula* were significantly correlated (*p* < 0.05) with the five enzyme activities in hepatopancreas and spleen, including 14 positive correlations and 13 negative correlations (Table S5). It is interesting that LZM was negatively correlated with the genus *Pirellula* in hepatopancreas (Figure 4a) but positively correlated with the genus *Pirellula* and *Mycobacterium* in the spleen (Figure 4b,c). GR was positively correlated with the genus *Mycobacterium* and *Pirellula* in the spleen (Figure 4d,e). In order to investigate the different effects of intestinal flora in hepatopancreas and spleen on the immune function of bighead carp, we compared all 14 positively correlated and 13 negatively correlated pairs. It is notable that LZM had opposite correlations with the enzyme activities of hepatopancreas and spleen (Table S5). Three pairs were found with consistent correlations in hepatopancreas and spleen, including *Mycobacterium* ~ GSH-PX (negative), *Pirellula* ~ GSH-PX (negative) and *Cetobacterium* ~ SOD (positive). The different non-specific immune functions of LZM in different tissues require further experiments to be explored.

## 4. Discussion

Fish intestinal microbes play an important role in inhibiting pathogenic microorganisms, the dominant flora in the intestine can protect the host from infection and damage of the complex microbes in the environment, the presence of certain intestinal microbial metabolites can stimulate the proliferation of intestinal epithelial cell and immune system response [32]. By analyzing the intestinal microbiota of healthy and diseased largemouth bronze gudgeon, Li et al. reported that the bacteria diversity was lower in diseased fish compared to healthy fish and that genus *Aeromonas* may be a key factor of causing infectious diseases of farmed fish [33]. Stagaman et al. analyzed the intestinal microorganisms of 68 wild type and 61 zebrafish lacking B or T cell receptor function, and showed that the immune could filter the composition of intestinal microorganisms, which might reflect the host immune status [34].

The mechanical, immune and biological barriers formed by the combination of animal intestinal flora and intestinal mucosa not only play an important role in maintaining the stability of the internal environment, but also effectively prevent the invasion of pathogenic substances and the displacement of bacterial endotoxin [35]. In this study, five indicators were considered to present the fish nonspecific immunity in the spleen and HP. Based on our analyses, LZM activity of bighead carp increased in group C compared to group B in the spleen after adding lactic acid bacteria, which is consistent with the findings by Son et al. in grouper blood [9], while in HP LZM, activity in groups B and C significantly decreased compared with group A (*p* < 0.001, Table 1). This intriguing finding might be consequences of the weakly acidic water after the addition of artificial feed or lactic acid bacteria and the HP is highly sensitive to water changes and may be suppressed to some extent. In addition, CAT and GR activities were changed in the spleen of bighead carp when after adding lactic acid bacteria alone (group C vs. group B), but there was no significant change in the HP, the results showed that lactic acid bacteria had different effects on CAT and GR activities in the spleen and HP of bighead carp. In a previous study, Lin et al. reported that lactic acid bacteria can significantly increase GSH-PX and SOD activities in the liver of mice compared with the control group [36]; however, very little research has been done in this area on fish. Our results showed that the GSH-PX and SOD activities decreased in HP but no change in the spleen of bighead carp when lactic acid bacteria were added to the feed, which can be explained by that there are certain differences between HP and spleen in the non-specific immune physiological functions of fish. Overall, although some enzyme activities were not observed with the same trends in HP and spleen of bighead carp when supplemented with lactic acid bacteria. However, according to the analysis of related enzyme activity index in the spleen, except CAT, all other enzyme activity indexes were increased or unchanged, these results roughly support that lactic acid bacteria are beneficial to improve the non-specific immune of bighead carp. Meanwhile, the effects and mechanisms of lactic acid bacteria in different fish organs may require further experiments to be explored.

It was reported that the gut specific microbial community of fish could be affected by the dietary manipulations [37,38,39]. However, there are few studies about the effects on gut microbiota of filter-feeding fish fed formulated feed. Earlier studies indicated the dominant phyla in the intestinal microbiota of healthy fish were *Proteobacteria* and *Firmicutes* [40,41], this is consistent with the overall results of our study. In addition, *Actinobacteria* was also found as a dominant phylum in the current study (Figure 2a), which is in accordance with that revealed for rainbow trout (Oncorhynchus mykiss) [42]. *Actinobacteria*, a phylum of Gram-positive bacteria, is an important secondary metabolite producer and plays a key role in animal intestines [43]. *Actinobacteria* have the ability to biosynthesize secondary metabolites as antibiotics against invasive pathogens [44]. Interestingly, we found that the relative abundance of *Actinobacteria* increased when the bighead carp were fed with formulated feed and/or lactic acid bacteria (Figure 2a). Likewise, Maria et al. found the phylum *Actinobacteria* was significantly increased when the colitis associated colon cancer model (C57BL/6 mice) was supplemented with probiotics [45]. While Ma et al. reported that the *Actinobacteria* as a dominant phylum in intestines of diseased fish compared to healthy controls [39], indicating that *Actinobacteria* may be useful biomarkers in immunocompromised transfused fish patients. Therefore, we believe that whether the phyla *Actinobacteria* is related to fish immunity needs to be further studied.

In this study, the genus *Cetobacterium* has the largest number of significant pair relationships in functional analysis (7 pairs, Table S5). It has been reported that genus *Cetobacterium* is widely distributed within the guts of freshwater fishes [46] and its prevalence is mainly contributing to the production of vitamin B12 in human [47]. Hence, *Cetobacterium* has been speculated to have a role in the synthesis of vitamin B12 in the fish gut [48]. However, in this study, vitamin B12 within the three groups was not investigated; further studies on this topic are needed. Because the abundance of *Cetobacterium* is positively correlated with the content of vitamin B12, supplementation of vitamin B12 in formulated fish feed may increase the abundance of *Cetobacterium* in group B and group C. In addition, as the dominant genera in LEfSe analyzed in group B and group C compared to group A, the *Cetobacterium* was revealed to be positively associated with SOD activity in HP and the spleen, and simultaneously suggested that the abundance of genus *Cetobacterium* may have a positive effect on the non-specific immune of fish. However, the genus *Cetobacterium* also showed a significant negative correlation with GSH-Px in the spleen; more microbial sequencing and functional activity study of intestinal microbiome in bighead carp are needed in the future.

In summary, the addition of lactic acid bacteria can significantly altered the composition of bighead carp intestinal flora, but the effects on non-specific immune indexes in HP and spleen were complex. It should be noted that this study did not carry out a variety of lactic acid bacteria experiments in bighead carp breeding, and the excess lactic acid bacteria may decrease the pH value of the water, thereby causing a certain stress to the HP. Therefore, more experiments are needed to verify this hypothesis. Currently, there are insufficient studies on the relationship between microorganisms and non-specific immunity in fish, thus, the research on the function and effect of fish intestinal microorganism needs to be strengthened.

## 5. Conclusions

In conclusion, we observed that the enzyme activities of bighead carp were modulated under different feeding strategies, especially when lactic acid was added to the feed. As a result of enzymatic analysis, compared with group B, the activities of LZM and GR in group C mainly increased in the spleen, while the activities of LZM, GSH-Px, and SOD decreased in the hepatopancreas. After the 16S rRNA gene sequencing data were analyzed for bighead carp with different strategies, we found higher bacterial α-diversities of the intestinal flora in the fish fed by formulated food with/without lactic acid bacteria, compared to group A. Moreover, we identified that three phyla—*Proteobacteria*, *Actinobacteria* and *Firmicutes*—were dominant in intestinal flora of bighead carp among the three groups. Importantly, when lactic acid bacteria were added to the feed, the relative abundance of *Actinobacteria* increased while the relative abundance of *Proteobacteria* and *Firmicutes* decreased. Correlation analysis identified 27 significantly correlated pairs (14 positive and 13 negative correlations) between the intestinal microbiome and the metabolic functions. In particular, LZM was found to affect the non-specific immune function of bighead carp in the spleen, and the genus *Cetobacterium* may be beneficial for non-specific immunity in fish. This study reveals the complex relationship between the gut microbes and non-specific immunity of bighead carp; the result will improve our understanding of the composition of bighead carp intestinal microbes under new rearing strategies.

## Figures and Tables

**Table 1:** Characteristics and non-specific immunity related enzyme index of bighead carp.

Characteristics/Tissue	Index	Group A (*n* = 9)	Group B (*n* = 15)	Group C (*n* = 15)	*p*-Value
Characteristics	Initial weight (g)	47.57 ± 6.08	52.40 ± 1.55	51.87 ± 2.13	NS
	Final weight (g)	77.85 ± 11.86	121.80 ± 2.90	120.54 ± 5.83	<0.01
	Weight gain rate (%)	63.40 ± 4.04	132.46 ± 1.75	132.34 ± 1.67	<0.001
	Growth rate (%)	50.47 ± 9.63	115.66 ± 2.37	114.44 ± 6.16	<0.001
	Survival rate (%)	100	100	100	NS
	Fatness (%)	1.97 ± 0.09	2.00 ± 0.09	2.00 ± 0.08	NS
	Visceral body ratio (%)	3.77 ± 0.54	6.17 ± 0.74	6.14 ± 0.64	<0.01
Hepatopancreas	LZM (ug/mL)	3.06 ± 0.39 ^a^	1.92 ± 0.28 ^b^	0.57 ± 0.02 ^c^	<0.001
	CAT (U/mgprot)	13.75 ± 2.37	11.00 ± 0.65	11.86 ± 0.34	NS
	GR (U/gprot)	18.36 ± 3.84	15.35 ± 3.72	18.46 ± 1.01	NS
	GSH-PX (U/mgprot)	1182.86 ± 65.99 ^a^	1162.77 ± 35.16 ^a^	760.89 ± 49.84 ^b^	<0.001
	SOD (U/mgprot)	34.85 ± 2.58 ^c^	130.29 ± 8.76 ^a^	86.57 ± 2.98 ^b^	<0.001
Spleen	LZM (ug/mL)	14.66 ± 0.31 ^b^	16.82 ± 1.14 ^b^	22.27 ± 2.22 ^a^	<0.01
	CAT (U/mgprot)	1.11 ± 0.06 ^b^	2.84 ± 0.30 ^a^	1.07 ± 0.29 ^b^	<0.001
	GR (U/gprot)	7.61 ± 0.75 ^c^	14.83 ± 1.43 ^b^	20.44 ± 0.78 ^a^	<0.001
	GSH-PX (U/mgprot)	670.89 ± 27.66 ^a^	449.05 ± 38.08 ^b^	452.16 ± 15.25 ^b^	<0.001
	SOD (U/mgprot)	381.52 ± 0.99 ^b^	449.27 ± 14.30 ^a^	450.89 ± 18.71 ^a^	<0.01

Note: values are expressed in mean ± SD. LZM, lysozyme; CAT, catalase; GR, glutathione reductase; GSH-PX, glutathione peroxidase; SOD, superoxide dismutase; HP, hepatopancreas; NS, not significant; ^a,^ ^b^ and ^c^ in the cells denote significant difference of group A, B and C, respectively, among the three groups (*p* < 0.05).

**Table 2:** Single sample diversity α-diversity analysis (mean +/− SD).

Group	OTUs	Chao	Shannon	Simpson	Coverage
A	1806 +/− 267	3111.11 +/− 543.80	4.20 +/− 0.47	0.1084 +/− 0.0659	0.9862 +/− 0.0029
B	1764 +/− 303	2844.00 +/− 562.98	4.52+/− 0.60	0.075 +/− 0.0499	0.9871 +/− 0.0027
C	2298 +/− 196	3739.67 +/− 315.29	5.16 +/− 0.291	0.0393 +/− 0.0137	0.9837 +/− 0.0026

## Data Availability

The 16S rRNA sequencing datasets of bighead carp intestinal bacteria was submitted to NCBI SRA (BioProject ID: PRJNA695396).

## Supplementary Materials

The following are available online at https://www.mdpi.com/article/10.3390/genes12060916/s1. Table S1. Enzyme activities in the hepatopancreas and spleen of bighead carp. Table S2. The status of sequencing data for each sample. Table S3. The bacteria community composition identified in the bighead with different feeding strategies on different taxonomic levels. Table S4. Differentially expressed bacteria identified in the intestinal flora of bighead carp with different feed strategies. Table S5. Pearson correlation analysis of enzyme activities and genera in the bighead carp from group C.
